# Effects of Olympic Weightlifting and Weightlifting‐Derivative Training on Vertical Jump Performance in Recreationally Trained Individuals: A Systematic Review and Network Meta‐Analysis

**DOI:** 10.1155/abb/6006181

**Published:** 2026-10-17

**Authors:** Bin Zhang

**Affiliations:** ^1^ Basic Teaching Department (Sports Department) Zhejiang Fashion Institute of Technology Ningbo China zjff.edu.cn

**Keywords:** countermovement jump, network meta-analysis, Olympic weightlifting, squat jump

## Abstract

**Background:**

Olympic weightlifting (OW) and weightlifting‐derivative training are widely used to improve explosive performance. However, the relative effectiveness of OW‐related training compared with other commonly used training interventions remains unclear. Therefore, this study used a systematic review and network meta‐analysis to compare and rank competing training interventions for vertical jump performance in recreationally trained individuals.

**Methods:**

PubMed, Web of Science Core Collection, and Scopus were searched from inception to February 3, 2026. Randomized controlled trials (RCTs) involving recreationally trained adults were included. Eight studies involving 233 participants (221 males and 12 females) with intervention durations ranging from 6 to 10 weeks were eligible. Primary outcomes included countermovement jump height (CMJ‐H), CMJ power (CMJ‐P), squat jump height (SJ‐H), and SJ power (SJ‐P). A fixed‐effect network meta‐analysis was conducted to compare these outcomes.

**Results:**

Eight RCTs involving 233 participants were included, and six studies (188 males) were eligible for network meta‐analysis. OW‐related training significantly improved CMJ height compared with control, plyometric training (PLYO), and traditional resistance training (RT) (mean difference [MD] = 2.21–2.54 cm). Similarly, OW‐related training significantly improved SJ height compared with control, PLYO, and RT (MD = 2.83–4.48 cm). For CMJ‐P, OW‐related training was superior to RT (MD = 3.22 W·kg^−1^, 95% confidence interval [CI]: 0.89–5.56). For SJ‐P, combined training (COMB) was superior to control (MD = 7.26 W·kg^−1^, 95% CI: 2.08–12.43). OW‐related training achieved the highest surface under the cumulative ranking curve (SUCRA) values for CMJ height (82.4%) and SJ height (90.2%).

**Conclusions:**

OW‐related training appears to be an effective strategy for improving vertical jump performance in recreationally trained individuals, particularly for CMJ height and SJ height. Based on the available evidence network, OW‐related training demonstrated relatively consistent positive effects across jump‐height outcomes and may be considered a viable training option for enhancing lower‐limb explosive performance.

## 1. Introduction

Olympic weightlifting (OW) training comprises the snatch, the clean, and jerk, and a variety of weightlifting derivatives (e.g., jump shrugs, power cleans, clean pulls, high pulls, and push presses). It is recognized as a training modality capable of eliciting high force production and movement velocity simultaneously [1, 2]. OW‐related exercises are characterized by coordinated triple extension of the hip, knee, and ankle joints, which facilitates rapid force transmission through the lower‐limb kinetic chain and enables explosive force production. Moreover, effective performance of these exercises requires substantial joint mobility, technical competency, and neuromuscular coordination, making OW training a highly skill‐dependent form of resistance exercise [3, 4]. From a biomechanical standpoint, OW‐related training may improve vertical jump performance through multiple mechanisms, including enhanced rate of force development (RFD), more effective utilization of the stretch–shortening cycle (SSC), and increased muscular power output [5, 6].

Vertical jump performance is a widely used measure of lower‐limb explosive power and has been shown to be closely related to athletic performance in numerous sports, particularly those requiring jumping, sprinting, and rapid changes of direction [7, 8]. In volleyball, for instance, greater jumping ability allows athletes to reach higher contact points during spiking and blocking, thereby improving their athletic performance and competitive success [9]. Beyond athletic populations, lower‐limb explosive power also plays an important role in physical function among the general population. Individuals with superior vertical jump performance often exhibit better balance control, greater gait stability, and enhanced physical function [10, 11]. Moreover, evidence suggests that better vertical jump performance is associated with enhanced sit‐to‐stand performance, improved dynamic balance, and superior overall physical functioning [12].

With the growing popularity of resistance training (RT) and fitness participation worldwide, an increasing number of recreationally trained individuals are undertaking structured exercise programs to improve their physical fitness and athletic performance [13]. Compared with elite athletes, these individuals typically possess some degree of training experience but generally demonstrate lower levels of technical proficiency and sport‐specific training development [14]. As a result, the physiological and performance adaptations induced by OW‐related training may differ from those observed in highly trained athletic populations. Furthermore, although OW remains a specialized competitive sport, its derivative exercises have been widely adopted in strength and conditioning practice and incorporated into high‐intensity functional training programs, including CrossFit [15, 16]. Therefore, evaluating the effectiveness of OW‐related training in recreationally trained individuals may provide valuable evidence for training prescription and practical application.

Previous systematic reviews and meta‐analyses have reported positive effects of OW‐related training on vertical jump performance, strength, and power development [17, 18]. Nevertheless, these findings have largely been derived from studies involving elite athletes or heterogeneous populations, with relatively limited evidence available for recreationally trained individuals. In addition, previous reviews have primarily relied on conventional pairwise meta‐analysis, which does not allow for the simultaneous comparison of multiple training modalities. In contrast, network meta‐analysis combines both direct and indirect evidence to compare and rank competing interventions within a single analytical framework, thereby providing more comprehensive and clinically relevant evidence for training practice [19]. Previous systematic reviews have demonstrated the effectiveness of OW‐related training; however, they were unable to establish the relative effectiveness and ranking of competing training modalities because they relied on conventional pairwise meta‐analysis.

Therefore, the aim of this study was to systematically evaluate and compare the effects of OW‐related training on vertical jump performance in recreationally trained individuals using a systematic review and network meta‐analysis approach. By synthesizing both direct and indirect evidence, we sought to determine the relative effectiveness of different training interventions and provide a more comprehensive evidence base for the practical application of OW‐related training among recreationally trained individuals. Based on previous findings, we hypothesized that OW‐related training would demonstrate superior effects on the vertical jump performance compared with other commonly used training modalities.

## 2. Information and Research Methods

This systematic review and network meta‐analysis was conducted in accordance with the PRISMA 2020 statement [20] and prospectively registered in the International Prospective Register of Systematic Reviews (PROSPERO) under Registration Number CRD42024543332.

### 2.1. Search Strategy

To systematically evaluate the effects of OW‐related training on vertical jump performance in recreationally trained individuals, a comprehensive literature search was conducted in PubMed, the Web of Science Core Collection, and Scopus from database inception to February 3, 2026.

The search strategy was developed around OW and weightlifting‐derivative training and combined terms related to training interventions and vertical jump performance. Search terms related to OW included “weightlifting,” “Olympic weightlifting,” “weight lifting,” “snatch,” “clean and jerk,” “power clean,” “hang clean,” “clean pull,” “snatch pull,” “high pull,” “jump shrug,” “mid‐thigh pull,” “push press,” and “weightlifting derivative.” Terms related to training interventions included “training,” “intervention,” “exercise program,” and “resistance training.” Outcome‐related terms included “vertical jump,” “countermovement jump,” “squat jump,” “jump height,” and “jump power.” These terms were combined using the Boolean operators “AND” and “OR,” as appropriate. The complete search strategies for each database are provided in Appendix S1.

### 2.2. Eligibility Criteria

Eligibility criteria were established according to the PICOS framework (population, intervention, comparator, outcomes, and study design).

#### 2.2.1. Inclusion Criteria

##### 2.2.1.1. Population

Eligible participants were healthy adults aged ≥ 18 years who were classified as recreationally trained individuals. Recreationally trained individuals were defined as non‐professional athletes with at least 6 months of structured fitness training, RT, high‐intensity functional training, or sport‐specific conditioning experience and a minimum training frequency of two sessions per week.

##### 2.2.1.2. Intervention

Eligible interventions consisted of OW‐related training, including exercises such as the snatch, clean and jerk, power clean, hang clean, clean pull, hang clean pull, jump shrug, mid‐thigh pull, push press, and other weightlifting‐derivative exercises. Studies were required to implement an intervention period of at least 6 weeks.

##### 2.2.1.3. Comparator

Comparators included nontraining control groups and other exercise training modalities used in the original studies. These interventions were included to construct the evidence network and support indirect comparisons within the network meta‐analysis.

##### 2.2.1.4. Outcomes

Studies were required to report vertical jump‐related outcomes, including squat jump (SJ), countermovement jump (CMJ), or other extractable measures of vertical jump height or jump power.

##### 2.2.1.5. Study Design

Only randomized controlled trials (RCTs) published as full‐text articles in peer‐reviewed English‐language journals were included.

#### 2.2.2. Exclusion Criteria

Studies were excluded if they (1) reported duplicate publications or overlapping datasets; (2) did not provide sufficient outcome data for quantitative synthesis, and such data could not be obtained from the corresponding authors; or (3) included interventions that did not meet the predefined definition of OW‐related training.

### 2.3. Study Selection

All retrieved records were imported into EndNote X9.1 for reference management and duplicate removal. Two reviewers independently screened the titles and abstracts of all records, followed by a full‐text assessment of potentially eligible studies. Any disagreements between the reviewers were resolved through discussion. If consensus could not be reached, a third reviewer was consulted to make the final decision.

### 2.4. Data Extraction

Data extraction was independently performed by two reviewers using a standardized Excel spreadsheet. The following information was extracted from each study: first author, publication year, participant characteristics, sample size, sex distribution, age, intervention duration, training frequency, intervention content, comparator condition, outcome measures, and pre‐ and postintervention means and standard deviations.

The primary outcomes were vertical jump height and vertical jump power. Specifically, data were extracted for CMJ height (CMJ‐H), CMJ power (CMJ‐P), SJ height (SJ‐H), and SJ power (SJ‐P). To ensure consistency across studies, jump height data were converted to centimeters (cm) when necessary. Jump power was preferentially expressed as values normalized to body mass (W · kg^−1^). When only absolute power values were reported and body mass data were available, the power values were normalized to body mass. If outcome data could not be obtained or calculated from the original publication, the corresponding authors were contacted to request additional information. If the required data remained unavailable, the corresponding outcome was excluded from the quantitative synthesis. Any disagreements during data extraction were resolved through discussion.

### 2.5. Reclassification of Interventions

To improve the interpretability and clarity of the evidence network, all interventions were reclassified according to their primary biomechanical characteristics, predominant training stimulus, exercise composition, and the proportion of OW‐related exercises within the training program.

The intervention nodes are defined as follows.

#### 2.5.1. OW‐Related Training (OW‐Related)

Interventions primarily consist of the snatch, clean and jerk, power clean, hang clean, clean pull, hang clean pull, jump shrug, mid‐thigh pull, push press, and other weightlifting‐derivative exercises.

#### 2.5.2. Plyometric Training (PLYO)

Interventions primarily consist of explosive jumping exercises, such as repeated jumps, bounding, hurdle jumps, drop jumps, and similar activities, with the training stimulus predominantly derived from SSC utilization.

#### 2.5.3. RT

Interventions primarily consisting of traditional strength‐training exercises that were not classified as OW‐related exercises, including squats, deadlifts, bench presses, pull‐ups, push‐ups, and other exercises, aimed primarily at increasing muscular strength or hypertrophy.

#### 2.5.4. Combined Training (COMB)

Interventions intentionally combine two or more training modalities within the same training program, such as OW‐related training combined with PLYO, RT, or high‐intensity functional training.

#### 2.5.5. Control

The participants who did not receive any structured training intervention or who maintained their usual daily activities.

For studies that included multiple training modalities, classification was based on the predominant training stimulus and the relative contribution of OW‐related exercises within the training program. Interventions were classified as OW‐related training when OW‐related exercises constituted the primary component and source of training stimulus. Interventions were classified as RT when they primarily consisted of traditional strength‐training exercises and did not include OW‐related movements. When two or more training modalities contributed substantially to the training stimulus and the intervention could not be reasonably assigned to OW‐related training, PLYO, or RT, it was classified as COMB.

### 2.6. Risk of Bias Assessment

The risk of bias of the included studies was independently assessed by two reviewers using the Cochrane risk of bias 2 (RoB 2) tool [21]. The following domains were evaluated: bias arising from the randomization process, bias due to deviations from intended interventions, bias due to missing outcome data, bias in the measurement of outcomes, and bias in the selection of the reported result.

According to the RoB 2 guidelines, each domain was rated as “Low Risk,” “Some Concerns,” or “High Risk.” Any disagreements between the reviewers were resolved through discussion, and a third reviewer was consulted when necessary. Final risk‐of‐bias judgments were determined through a consensus process.

### 2.7. Statistical Analysis

Statistical analyses were performed using R (Version 4.4.2; R Foundation for Statistical Computing, Vienna, Austria) with the netmeta package. The network meta‐analysis was conducted using a contrast‐based fixed‐effect model. As all outcomes were continuous variables, intervention effects were expressed as mean differences (MDs) with 95% confidence intervals (CIs).

Network plots were generated to illustrate the direct comparisons among interventions. Nodes represented different intervention categories, with the node size proportional to the total sample size for each intervention. Edges represented direct comparisons, and edge thickness reflected the number of studies contributing to each comparison. The transitivity assumption was assessed by examining the similarity of potential effect modifiers across studies, including participant age, sex distribution, training background, intervention duration, training frequency, and outcome assessment methods. For comparisons in which direct and indirect evidence could be separated, consistency was evaluated using the node‐splitting approach. A *p* value >0.05 was considered indicative of no significant inconsistency between direct and indirect evidence.

Interventions were ranked using rank probabilities and the surface under the cumulative ranking curve (SUCRA). Rank probabilities were estimated from the fitted treatment effects and their variance–covariance matrix using 50,000 simulations. Given the limited number of included studies and the sparse structure of some outcome networks, the ranking results were interpreted as exploratory findings.

## 3. Results

### 3.1. Characteristics of Included Studies

A total of 2282 records were identified through database searching, including 880 records from PubMed, 895 from the Web of Science Core Collection, and 507 from Scopus. The complete search strategies for each database are provided in Appendix S1. After removing 266 duplicate records, 2016 records remained for title and abstract screening. Of these, 92 articles were assessed for full‐text eligibility. Following a full‐text review, 78 studies were excluded because they did not meet the predefined inclusion criteria regarding intervention characteristics, outcome measures, study design, or participant characteristics. In addition, manual screening of the reference lists of eligible studies identified two further studies that met the inclusion criteria. Ultimately, eight RCTs were included in the review [22–29]. The study selection process is presented in Figure 1.

**Figure 1:**
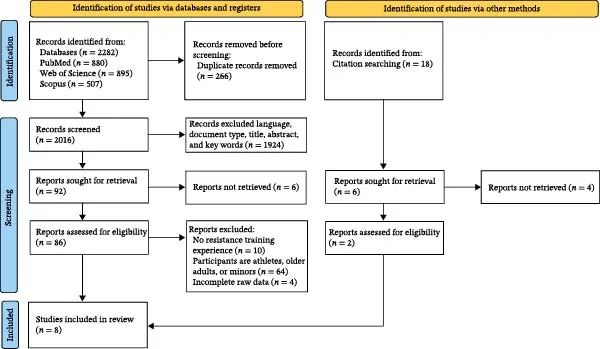
PRISMA 2020 flow diagram of study selection.

The included studies involved a total of 233 recreationally trained individuals, including 221 males and 12 females. Sample sizes ranged from 20 to 45 participants, intervention durations ranged from 6 to 10 weeks, and training frequency was typically two–four sessions per week. According to the predefined classification criteria, the included studies comprised the following intervention nodes: OW‐related training, PLYO, RT, COMB, and control.

All studies used CMJ and/or SJ as measures of vertical jump performance and reported jump height and/or jump power outcomes. Detailed characteristics of the participants and interventions are presented in Table S1 (Appendix S2).

### 3.2. Risk of Bias Assessment

The risk‐of‐bias assessment results for the eight included RCTs are presented in Figure 2. One study was judged as having a low risk of bias, whereas the remaining seven studies were rated as having some concerns. The most common sources of bias were insufficient reporting of the randomization process, lack of information regarding allocation concealment, inadequate reporting of assessor blinding, and the absence of a prespecified protocol or statistical analysis plan. In contrast, the risk of bias arising from missing outcome data was generally low as most studies reported complete follow‐up and outcome data. Overall, no study was judged to have a high risk of bias, although most studies were classified as having some concerns.

**Figure 2:**
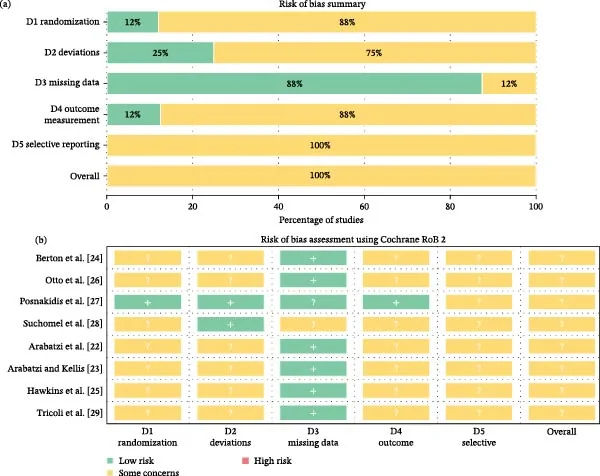
The results of the risk of bias assessment. (a) Risk of bias graph. (b) Risk of bias summary.

### 3.3. Network Structure

Among the included studies, both the HIFT‐P and HIFT‐C groups in Georgios et al. (2022) were classified as COMB, whereas the CATCH, PULL, and OL groups in Timothy et al. (2020) were classified as OW‐related. Consequently, these two studies only contributed within‐node comparisons and did not provide direct comparisons between different intervention nodes; therefore, they were not included in the network meta‐analysis.

Ultimately, six studies provided direct comparisons between different intervention nodes and were included in the network meta‐analysis, involving a total of 188 male participants. The network structures for each outcome are presented in Figure 3.

**Figure 3:**
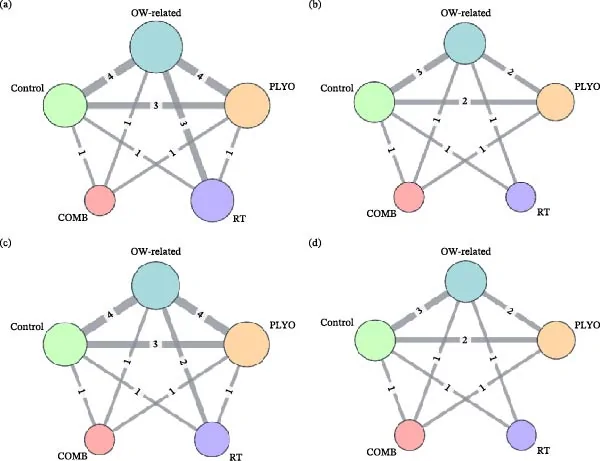
Network graphs of training interventions. (a) CMJ‐height, (b) CMJ‐power, (c) SJ‐height, and (d) SJ‐power. CMJ, countermovement jump; COMB, combined training; control, no‐training control; OW‐related training, Olympic weightlifting and weightlifting‐derivative training; PLYO, plyometric training; RT, resistance training; SJ, squat jump.

### 3.4. Network Meta‐Analysis Results

The results of the network meta‐analysis for CMJ‐H, CMJ‐P, SJ‐H, and SJ‐P are presented in Table 1. Effect estimates are expressed as MDs with 95% CIs.

**Table 1:** League tables of network meta‐analysis for CMJ height, CMJ power, SJ height, and SJ power.

Outcome measure	Treatment	Control	OW‐related	PLYO	RT	COMB
CMJ‐height	Control	—	−2.21 (−4.41, −0.01)	−0.14 (−2.49, 2.20)	0.33 (−2.31, 2.96)	−3.43 (−9.43, 2.57)
	OW‐related	2.21 (0.01, 4.41)	—	2.07 (0.09, 4.05)	2.54 (0.64, 4.43)	−1.22 (−7.20, 4.76)
	PLYO	0.14 (−2.20, 2.49)	−2.07 (−4.05, −0.09)	—	0.47 (−2.16, 3.10)	−3.29 (−9.28, 2.70)
	RT	−0.33 (−2.96, 2.31)	−2.54 (−4.43, −0.64)	−0.47 (−3.10, 2.16)	—	−3.76 (−9.97, 2.46)
	COMB	3.43 (−2.57, 9.43)	1.22 (−4.76, 7.20)	3.29 (−2.70, 9.28)	3.76 (−2.46, 9.97)	—
CMJ‐power	Control	—	−0.49 (−2.78, 1.79)	−0.56 (−3.84, 2.72)	2.73 (0.65, 4.81)	1.51 (−4.84, 7.87)
	OW‐related	0.49 (−1.79, 2.78)	—	−0.07 (−3.23, 3.10)	3.22 (0.89, 5.56)	2.01 (−4.22, 8.23)
	PLYO	0.56 (−2.72, 3.84)	0.07 (−3.10, 3.23)	—	3.29 (−0.28, 6.86)	2.07 (−4.07, 8.22)
	RT	−2.73 (−4.81, −0.65)	−3.22 (−5.56, −0.89)	−3.29 (−6.86, 0.28)	—	−1.22 (−7.70, 5.27)
	COMB	−1.51 (−7.87, 4.84)	−2.01 (−8.23, 4.22)	−2.07 (−8.22, 4.07)	1.22 (−5.27, 7.70)	—
SJ‐height	Control	—	−3.01 (−5.11, −0.91)	−0.18 (−2.37, 2.01)	1.47 (−1.47, 4.42)	−2.25 (−7.37, 2.87)
	OW‐related	3.01 (0.91, 5.11)	—	2.83 (0.89, 4.76)	4.48 (1.60, 7.37)	0.76 (−4.39, 5.90)
	PLYO	0.18 (−2.01, 2.37)	−2.83 (−4.76, −0.89)	—	1.66 (−1.56, 4.87)	−2.07 (−7.30, 3.16)
	RT	−1.47 (−4.42, 1.47)	−4.48 (−7.37, −1.60)	−1.66 (−4.87, 1.56)	—	−3.73 (−9.44, 1.99)
	COMB	2.25 (−2.87, 7.37)	−0.76 (−5.90, 4.39)	2.07 (−3.16, 7.30)	3.73 (−1.99, 9.44)	—
SJ‐power	Control	—	−2.43 (−5.04, 0.19)	−1.68 (−4.85, 1.49)	−4.38 (−7.26, −1.49)	−7.26 (−12.43, −2.08)
	OW‐related	2.43 (−0.19, 5.04)	—	0.74 (−2.09, 3.57)	−1.95 (−5.54, 1.64)	−4.83 (−9.90, 0.24)
	PLYO	1.68 (−1.49, 4.85)	−0.74 (−3.57, 2.09)	—	−2.69 (−6.80, 1.42)	−5.57 (−10.65, −0.49)
	RT	4.38 (1.49, 7.26)	1.95 (−1.64, 5.54)	2.69 (−1.42, 6.80)	—	−2.88 (−8.69, 2.93)
	COMB	7.26 (2.08, 12.43)	4.83 (−0.24, 9.90)	5.57 (0.49, 10.65)	2.88 (−2.93, 8.69)	—

*Note:* Values are presented as mean differences (MDs) with 95% confidence intervals (CIs). COMB, combined training; control, no‐training control; OW‐related training, Olympic weightlifting and weightlifting‐derivative training; PLYO, plyometric training.

Abbreviations: CMJ, countermovement jump; RT, resistance training; SJ, squat jump.

The ranking probabilities of the different training interventions for CMJ‐H, CMJ‐P, SJ‐H, and SJ‐P are presented in Figure 4, and the corresponding SUCRA values are provided in Table S6. For CMJ‐H, COMB showed the highest probability of being ranked first, whereas OW‐related training achieved the highest SUCRA value (82.4%). For CMJ‐P, OW‐related training and PLYO showed the highest SUCRA values (72.0% and 71.5%, respectively). For SJ‐H, OW‐related training achieved the highest SUCRA value (90.2%), whereas COMB achieved the highest SUCRA value for SJ‐P (94.7%).

**Figure 4:**
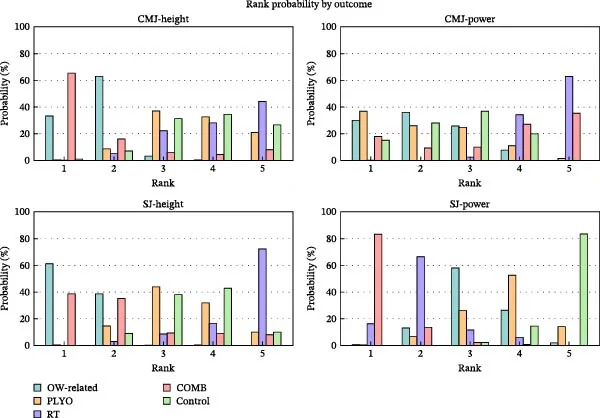
Rank probability distributions of intervention nodes across four vertical jump outcomes.

The node‐splitting analyses presented in Appendix S3 (Tables S2–S5) showed no significant inconsistency between direct and indirect evidence for CMJ‐H, CMJ‐P, SJ‐H, and SJ‐P.

#### 3.4.1. CMJ‐H

For CMJ‐H, OW‐related training was associated with significantly greater improvements than Control (MD = 2.21 cm, 95% CI: 0.01–4.41), PLYO (MD = 2.07 cm, 95% CI: 0.09–4.05), and RT (MD = 2.54 cm, 95% CI: 0.64–4.43). No significant difference was observed between OW‐related training and COMB (MD = 1.22 cm, 95% CI: −4.76 to 7.20).

The ranking probability analysis indicated that COMB had the highest probability of being ranked first (65.4%), followed by OW‐related training (33.4%). However, OW‐related training achieved the highest SUCRA value (82.4%), followed closely by COMB (81.6%).

#### 3.4.2. CMJ‐P

For CMJ‐P, OW‐related training resulted in significantly greater improvements than RT (MD = 3.22 W · kg^−1^, 95% CI: 0.89–5.56). In addition, a significant difference was observed between control and RT (MD = 2.73 W · kg^−1^, 95% CI: 0.65–4.81). No significant differences were found between OW‐related training and control, between PLYO and control, or between PLYO and OW‐related training.

The ranking probability analysis indicated that PLYO had the highest probability of being ranked first (36.8%), followed by OW‐related training (29.9%) and COMB (18.2%). OW‐related training (72.0%) and PLYO (71.5%) achieved the highest SUCRA values, indicating similar overall ranking performance.

#### 3.4.3. SJ‐H

For SJ‐H, compared with Control (MD = 3.01 cm, 95% CI: 0.91–5.11), PLYO (MD = 2.83 cm, 95% CI: 0.89–4.76), and RT (MD = 4.48 cm, 95% CI: 1.60–7.37), OW‐related training resulted in significantly greater improvements. No significant difference was observed between OW‐related training and COMB (MD = 0.76 cm, 95% CI: −4.39 to 5.90).

The ranking probability analysis indicated that OW‐related training had the highest probability of being ranked first (61.1%), followed by COMB (38.7%). In addition, OW‐related training achieved the highest SUCRA value (90.2%).

#### 3.4.4. SJ‐P

For SJ‐P, COMB resulted in significantly greater improvements than Control (MD = 7.26 W · kg^−1^, 95% CI: 2.08–12.43) and PLYO (MD = 5.57 W · kg^−1^, 95% CI: 0.49–10.65). RT also resulted in significantly greater improvements than control (MD = 4.38 W · kg^−1^, 95% CI: 1.49–7.26). No significant differences were observed between OW‐related training and control or between OW‐related training and PLYO.

The ranking probability analysis indicated that COMB had the highest probability of being ranked first (83.2%), followed by RT (15.9%). In addition, COMB achieved the highest SUCRA value (94.7%).

## 4. Discussion

This systematic review and network meta‐analysis examined the effects of OW‐related training on the vertical jump performance in recreationally trained individuals. Using the evidence network generated from the included studies, we further explored the relative effects of OW‐related training compared with those of other comparator interventions. Overall, the findings suggest that OW‐related training was associated with relatively consistent improvements in CMJ‐H and SJ‐H.

The present findings are generally consistent with those reported in previous systematic reviews and meta‐analyses. Hackett et al. [30] reported that OW training could significantly improve vertical jump performance and produced training adaptations comparable to those observed with traditional RT. Subsequently, García‐Valverde et al. [31], in a meta‐analysis including 34 studies, found that weightlifting‐based training effectively enhanced CMJ performance and promoted lower‐limb power development. Together, these studies support the effectiveness of OW‐related training for improving the vertical jump performance. Extending this evidence, the present network meta‐analysis provides additional insight into the relative effects of OW‐related training within a network of comparator interventions in recreationally trained individuals.

The beneficial effects of OW‐related training on vertical jump performance are supported by well‐established biomechanical and movement‐specific mechanisms. OW and weightlifting‐derivative exercises require athletes to generate force rapidly and explosively within a very short period of time while producing substantial vertical ground reaction forces through coordinated extension of the hip, knee, and ankle joints [32]. This movement pattern closely resembles the triple‐extension action that characterizes vertical jumping and may therefore facilitate a strong transfer of training adaptations to jump performance [33]. Compared with traditional RT, which primarily emphasizes the development of maximal strength, OW‐related training places greater demands on the simultaneous expression of force and velocity, resulting in higher power output and RFD [34]. Because vertical jump performance depends largely on the ability to generate force rapidly within a limited time frame, improvements in RFD are considered one of the primary mechanisms underlying enhancements in jumping performance [35, 36].

From a neuromuscular adaptation perspective, OW‐related training may enhance explosive performance by improving motor unit recruitment, increasing the activation of high‐threshold motor units, and enhancing intermuscular coordination [5]. In addition, the complex multijoint nature of OW‐related movements may improve the neuromuscular system’s ability to coordinate and control rapid force‐production tasks, thereby contributing to enhanced lower‐limb explosive performance [2, 37]. Therefore, compared with traditional RT, which primarily emphasizes maximal strength development, OW‐related training may simultaneously promote the development of strength, velocity, and neuromuscular coordination, making it particularly relevant to the demands of explosive athletic movements [34, 38].

The concurrent improvements observed in both CMJ‐H and SJ‐H further support this interpretation. CMJ includes a distinct countermovement phase and is therefore more dependent on SSC utilization, whereas SJ largely minimizes SSC contribution and more directly reflects concentric force‐producing capability and voluntary power output [39, 40]. Theoretically, if a training intervention primarily enhances SSC utilization, greater improvements would be expected in CMJ than in SJ [41]. However, in the present study, OW‐related training demonstrated favorable effects on both outcomes. This finding suggests that the observed training adaptations may not be attributable solely to enhanced SSC utilization but may also reflect broader improvements in lower‐limb explosive performance and neuromuscular function [5].

It is noteworthy that although PLYO is widely recognized as an effective strategy for improving jump performance, its benefits are generally attributed to enhanced SSC utilization and reactive strength [42, 43]. However, OW‐related training is also characterized by high movement velocities and explosive force production demands, both of which can improve the neuromuscular system’s ability to generate force rapidly [6]. Furthermore, several weightlifting‐derivative exercises exhibit pronounced ballistic characteristics and can develop force–velocity qualities under relatively high loading conditions [5, 44]. Consequently, the training adaptations induced by OW‐related training and PLYO may partially overlap. This shared physiological and neuromuscular basis may help explain why no consistent differences were observed between the two interventions in the present study.

However, the patterns observed for jump height and jump power outcomes were not entirely consistent in the present study. Compared with jump height, power‐related outcomes may be more susceptible to differences in testing equipment, calculation procedures, and data standardization methods. In addition, several studies did not report body mass‐normalized power values, which reduced the number of studies available for quantitative synthesis. Therefore, the findings related to jump power should be interpreted with caution and require further confirmation from future studies employing standardized assessment and reporting procedures.

Several limitations of the present network meta‐analysis should be acknowledged. First, only eight RCTs were included, and some outcome networks were relatively sparse, which may have reduced the statistical power of indirect comparisons and the precision of intervention rankings. Therefore, the SUCRA rankings and indirect comparisons should be considered exploratory findings and interpreted cautiously. Second, differences existed across studies in intervention duration, training frequency, training content, and outcome assessment methods. For example, some studies did not clearly report whether arm swing was permitted during vertical jump testing, which may have contributed to additional variability in the results [45, 46]. In addition, female participants were underrepresented in the available literature, which may limit the external validity of the current findings. Therefore, future studies involving more diverse populations, particularly female participants, are needed to further clarify the effects of OW‐related training across different populations. Furthermore, although no major violations of the transitivity assumption were identified, residual differences in training background, RT experience, and technical proficiency may still have influenced the validity of indirect comparisons [47]. Finally, due to the limited number of available studies, publication bias was not formally assessed, and the certainty of evidence was not evaluated using the GRADE or CINeMA. Although the present findings provide comparative evidence among different training interventions, the lack of a formal certainty‐of‐evidence assessment should be considered when interpreting the treatment effects and intervention rankings.

## 5. Conclusions

The findings of this network meta‐analysis suggest that OW‐related training may be an effective strategy for improving the vertical jump performance in recreationally trained individuals. Based on the evidence network derived from the included studies, OW‐related training demonstrated relatively consistent positive effects on both CMJ‐H and SJ‐H and showed favorable effects compared with several comparator interventions for selected outcomes. Overall, OW‐related training may be considered a viable training option for enhancing lower‐limb explosive performance and vertical jump ability in recreationally trained individuals. However, the effectiveness of OW‐related training may be influenced by participants’ training background and technical proficiency, and these factors should be considered when implementing OW‐related exercises in practice.

**Nomenclature**
CI: Confidence interval
CMJ: Countermovement jump
CMJ‐H: Countermovement jump height
CMJ‐P: Countermovement jump power
COMB: Combined training
MD: Mean difference
OW: Olympic weightlifting
PLYO: Plyometric training
RCT: Randomized controlled trial
RFD: Rate of force development
RoB 2: Risk of bias 2
RT: Traditional resistance training
SJ: Squat jump
SJ‐H: Squat jump height
SJ‐P: Squat jump power
SSC: Stretch–shortening cycle
SUCRA: Surface under the cumulative ranking curve.

## Funding

This study was sponsored by the Zhejiang Provincial Philosophy and Social Sciences Planning Project (Grant 26NDJC084YBM).

## Conflicts of Interest

The author declares no conflicts of interest.

## Supporting Information

Additional supporting information can be found online in the Supporting Information section.

## Supporting information


**Supporting Information** The following Supporting Information is available online at the publisher’s website: Appendix S1: Detailed search strategy. Search strategies used for PubMed, Web of Science Core Collection, and Scopus. Appendix S2: Characteristics of included studies. Table S1: Characteristics and intervention classifications of the included studies. Appendix S3: Node‐splitting analysis results. Tables S2–S5: Node‐splitting analyses for CMJ height, CMJ power, SJ height, and SJ power. Appendix S4: SUCRA analysis results. Table S6: SUCRA values and intervention rankings for CMJ height, CMJ power, SJ height, and SJ power.

## Data Availability

The data that support the findings of this study are available upon request from the corresponding author. The data are not publicly available due to privacy or ethical restrictions.
