# Supplementary material for: Effects of Olympic Weightlifting and Weightlifting‐Derivative Training on Vertical Jump Performance in Recreationally Trained Individuals: A Systematic Review and Network Meta‐Analysis
Source: Appl Bionics Biomech. 2026 Oct 17;2026:6006181. doi: 10.1155/abb/6006181 (PMC13675844; doi:10.1155/abb/6006181)
Supplement: Supplementary file 1 [file ABB-2026-6006181-s001.docx]

**Supplementary Information for:**

**Effects of Olympic Weightlifting and Weightlifting-Derivative Training on Vertical Jump Performance in recreationally trained individuals: A Systematic Review and Network Meta-Analysis**

**Appendix S1. Detailed Search Strategy**

The literature search was conducted in PubMed, Web of Science Core Collection, and Scopus from database inception to 3 February 2026. The search strategy was designed to be sensitive at the database-search stage. Therefore, terms related to Olympic weightlifting and weightlifting-derived exercises were combined with broad terms for training, exercise, performance, strength, power, and jumping outcomes. Boolean operators "OR" and "AND" were used to combine search terms.

The database search identified a total of 2,282 records: 880 from PubMed, 895 from Web of Science Core Collection, and 507 from Scopus.

1. **PubMed**

Records identified: 880

("Weight Lifting"[Mesh]

OR weightlifting[Title/Abstract]

OR "weight lifting"[Title/Abstract]

OR "Olympic weightlifting"[Title/Abstract]

OR "Olympic weight lifting"[Title/Abstract]

OR "weightlift*"[Title/Abstract]

OR snatch[Title/Abstract]

OR snatches[Title/Abstract]

OR "clean and jerk"[Title/Abstract]

OR "clean & jerk"[Title/Abstract]

OR "power clean"[Title/Abstract]

OR "hang clean"[Title/Abstract]

OR "clean pull"[Title/Abstract]

OR "snatch pull"[Title/Abstract]

OR "high pull"[Title/Abstract]

OR "jump shrug"[Title/Abstract]

OR "mid-thigh pull"[Title/Abstract]

OR "mid thigh pull"[Title/Abstract]

OR "push press"[Title/Abstract]

OR "weightlifting derivative"[Title/Abstract]

OR "weightlifting derivatives"[Title/Abstract]

OR "weight lifting derivative"[Title/Abstract]

OR "weight lifting derivatives"[Title/Abstract]

OR "weightlifting exercise"[Title/Abstract]

OR "weightlifting exercises"[Title/Abstract]

OR "Olympic lifting"[Title/Abstract]

OR "Olympic lift"[Title/Abstract])

AND

( "Exercise"[Mesh]

OR "Exercise Therapy"[Mesh]

OR "Resistance Training"[Mesh]

OR "Athletic Performance"[Mesh]

OR training[Title/Abstract]

OR train*[Title/Abstract]

OR intervention[Title/Abstract]

OR interventions[Title/Abstract]

OR exercise[Title/Abstract]

OR exercises[Title/Abstract]

OR "exercise program"[Title/Abstract]

OR "exercise programme"[Title/Abstract]

OR "exercise programs"[Title/Abstract]

OR "exercise programmes"[Title/Abstract]

OR "resistance training"[Title/Abstract]

OR "strength training"[Title/Abstract]

OR "power training"[Title/Abstract]

OR "plyometric training"[Title/Abstract]

OR "jump training"[Title/Abstract]

OR program[Title/Abstract]

OR programme[Title/Abstract]

OR protocol[Title/Abstract]

OR performance[Title/Abstract]

OR adaptation[Title/Abstract]

OR adaptations[Title/Abstract])

AND

( "Jumping"[Mesh]

OR jump*[Title/Abstract]

OR "vertical jump"[Title/Abstract]

OR "vertical jumping"[Title/Abstract]

OR "countermovement jump"[Title/Abstract]

OR "counter movement jump"[Title/Abstract]

OR CMJ[Title/Abstract]

OR "squat jump"[Title/Abstract]

OR SJ[Title/Abstract]

OR "drop jump"[Title/Abstract]

OR "jump height"[Title/Abstract]

OR "jump power"[Title/Abstract]

OR "jump performance"[Title/Abstract]

OR "lower-body power"[Title/Abstract]

OR "lower body power"[Title/Abstract]

OR "muscle power"[Title/Abstract]

OR "explosive strength"[Title/Abstract]

OR "explosive performance"[Title/Abstract]

OR "power output"[Title/Abstract)

2. **Web of Science Core Collection**

Records identified: 895

TS=( (weightlifting

OR "weight lifting"

OR "Olympic weightlifting"

OR "Olympic weight lifting"

OR weightlift*

OR snatch

OR snatches

OR "clean and jerk"

OR "clean & jerk"

OR "power clean"

OR "hang clean"

OR "clean pull"

OR "snatch pull"

OR "high pull"

OR "jump shrug"

OR "mid-thigh pull"

OR "mid thigh pull"

OR "push press"

OR "weightlifting derivative"

OR "weightlifting derivatives"

OR "weight lifting derivative"

OR "weight lifting derivatives"

OR "weightlifting exercise"

OR "weightlifting exercises"

OR "Olympic lifting"

OR "Olympic lift" )

AND

(training

OR train*

OR intervention*

OR exercise*

OR "exercise program"

OR "exercise programme"

OR "exercise programs"

OR "exercise programmes"

OR "resistance training"

OR "strength training"

OR "power training"

OR "plyometric training"

OR "jump training"

OR program

OR programme

OR protocol

OR performance

OR adaptation* )

AND

(jump*

OR "vertical jump"

OR "vertical jumping"

OR "countermovement jump"

OR "counter movement jump"

OR CMJ

OR "squat jump"

OR SJ

OR "drop jump"

OR "jump height"

OR "jump power"

OR "jump performance"

OR "lower-body power"

OR "lower body power"

OR "muscle power"

OR "explosive strength"

OR "explosive performance"

OR "power output"))

3. **Scopus**

Records identified: 507

TITLE-ABS-KEY ((weightlifting

OR "weight lifting"

OR "Olympic weightlifting"

OR "Olympic weight lifting"

OR weightlift*

OR snatch

OR snatches

OR "clean and jerk"

OR "clean & jerk"

OR "power clean"

OR "hang clean"

OR "clean pull"

OR "snatch pull"

OR "high pull"

OR "jump shrug"

OR "mid-thigh pull"

OR "mid thigh pull"

OR "push press"

OR "weightlifting derivative"

OR "weightlifting derivatives"

OR "weight lifting derivative"

OR "weight lifting derivatives"

OR "weightlifting exercise"

OR "weightlifting exercises"

OR "Olympic lifting"

OR "Olympic lift" )

AND

(training

OR train*

OR intervention*

OR exercise*

OR "exercise program"

OR "exercise programme"

OR "exercise programs"

OR "exercise programmes"

OR "resistance training"

OR "strength training"

OR "power training"

OR "plyometric training"

OR "jump training"

OR program

OR programme

OR protocol

OR performance

OR adaptation*)

AND

(jump*

OR "vertical jump"

OR "vertical jumping"

OR "countermovement jump"

OR "counter movement jump"

OR CMJ

OR "squat jump"

OR SJ

OR "drop jump"

OR "jump height"

OR "jump power"

OR "jump performance"

OR "lower-body power"

OR "lower body power"

OR "muscle power"

OR "explosive strength"

OR "explosive performance"

OR "power output" ))

The search results were exported to EndNote X9.1 for duplicate removal and screening.

**Appendix S2. Characteristics of included studies**

Table S1 presents the detailed characteristics of the included studies, including participant demographics, intervention protocols, training duration and frequency, comparator conditions, outcome measures, and key findings.

**Table S1. Characteristics of participants and interventions in the included studies**

| **Study** | **Participants (M/F)** | **Age (years)** | **Intervention Groups (Original)** | **Reclassified Node** | **Duration** | **Frequency** | **Outcomes** |
| --- | --- | --- | --- | --- | --- | --- | --- |
| Ricardo 2022[1] | 45/0 | WL: 24.1 ± 4.2; | WL (n=15): High pull from the knee, Power clean from the knee, Mid-thigh clean pull; | OW-related | 8 weeks | WL: 3 days/week | CMJ Height, CMJ Power,  SJ Height,  SJ Power |
|  |  | Plyo: 23.9 ± 4.7; | Plyo (n=15): Bounce drop jump,Double-leg hurdle hops,Horizontal jumps; | PLYO |  | Plyo: 3 days/week |  |
|  |  | CG: 24.3 ± 3.2 | CG (n=15): no structured training | Control |  | CG: N/A |  |
| William 2012[2] | 30/0 | WL: 22.92 ± 1.98; | WL (n=13): high pulls, power cleans, back squats; | OW-related | 6 weeks | WL: 2 days/week | CMJ Height |
|  |  | KB: 22.76 ± 1.86 | KB (n=17): kettlebell swings, accelerated swings, goblet squats | RT |  | KB: 2 days/week |  |
| Georgios 2022[3] | 8/12 | HIFT-P: 30 ± 4; | HIFT-P (n=10): clean-and-press, box jump, TRX chest press, wall ball throws, burpees, repeated 10 m sprints...; | COMB | 8 weeks | HIFT-P: 3 days/week | CMJ Height,  SJ Height |
|  |  | HIFT-C: 30 ± 4 | HIFT-C (n=10): clean-and-press, box jump, TRX chest press, wall ball throws, burpees, repeated 10 m sprints... | COMB |  | HIFT-C: 3 days/week |  |
| Timothy 2020[4] | 25/0 | CATCH: 22.1 ±3.3; | CATCH (n=8): Traditional Exercises and PC from Floor,Mid-Thigh PC, CM PC, Hang PC; | OW-related | 10 weeks | CATCH: 3 days/week | CMJ Height, CMJ Power,  SJ Height,  SJ Power |
|  |  | PULL: 22.2 ± 2.3; | PULL (n=9): Traditional Exercises and CP from Floor,Mid-Thigh Pull,CM Shrug, Hang High Pull,Jump Shrug; | OW-related |  | PULL: 3 days/week |  |
|  |  | OL: 22.5 ± 1.2 | OL (n=8): Traditional Exercises and CP from Floor,Mid-Thigh Pull,HangHigh Pull,Jump Shrug | OW-related |  | OL: 3 days/week |  |
| Fotini 2010[5] | 36/0 | WL: 20.3 ± 2; | WL (n=9): Power clean,Snatch,Clean and jerk,High pull,Half-squat; | OW-related | 8 weeks | WL: 3 days/week | CMJ Height, CMJ Power,  SJ Height,  SJ Power |
|  |  | PL: 20.3 ± 2; | PL (n=9): Double-leg hurdle hops,Alternated single-leg hurdle hops,Double-leg hops,Half-squat; | PLYO |  | PL: 3 days/week |  |
|  |  | WP: 20.3 ± 2; | WP (n=10): Power clean ,Snatch ,Clean and jerk ,High pull,Half-squat,Double-leg hurdle hop,Alternated s...; | COMB |  | WP: 3 days/week |  |
|  |  | C: 20.3 ± 2 | C (n=8): Control/no structured training | Control |  | C: N/A |  |
| Fotini 2012[6] | 26/0 | OL: 20.3 ± 2; | OL (n=9): Power clean,Snatch,Clean and jerk,High pull,Half-squat; | OW-related | 8 weeks | OL: 3 days/week | CMJ Height, CMJ Power,  SJ Height,  SJ Power |
|  |  | TW: 20.3 ± 2; | TW (n=9): Leg press,Leg curl,Leg extension,Bench press,Half-squat; | RT |  | TW: 3 days/week |  |
|  |  | C: 20.3 ± 2 | C (n=8): Control/no structured training | Control |  | C: N/A |  |
| Sheldon 2009[7] | 29/0 | WT: 21.5 ± 12.5; | WT (n=10): Squats,Lunges,Bent-over row,Pullover,Upright row,Squats,Lunges,Prone pull-ups,Bench press,Go...; | RT | 8 weeks | WT: 3 days/week | CMJ Height, CMJ Power,  SJ Height,  SJ Power |
|  |  | Plyo: 21.5 ± 12.5; | Plyo (n=10): Jumps in place, Squat jumps, A-skips, Single-leg hops, Double-leg hops, Double-leg hops, Jum...; | PLYO |  | Plyo: 3 days/week |  |
|  |  | WL: 21.5 ± 12.5 | WL (n=9): High pull,Hang clean,Power push (front),Snatch balance,Front squats,Hang clean (below knee)... | OW-related |  | WL: 3 days/week |  |
| Valmon 2005[8] | 22/0 | WL: 22.0 ± 1.5; | WL (n=7): high pull,high pull,clean and jerk; | OW-related | 8 weeks | WL: 3 days/week | CMJ Height,  SJ Height |
|  |  | VJ: 22.0 ± 1.5; | VJ (n=8): double-leg hurdle hops, alternated single-leg hurdle hops, single-leg hurdle hops,40-cm drop...; | PLYO |  | VJ: 3 days/week |  |
|  |  | C: 22.0 ± 1.5 | C (n=7): Control/no structured training | Control |  | C: N/A |  |

M = male; F = female; OW = Olympic weightlifting; OW-related = Olympic weightlifting and weightlifting-derivative training; PLYO = plyometric or jump training; RT = non-Olympic-weightlifting resistance training; COMB = combined or complex training; CMJ = countermovement jump; SJ = squat jump; HIFT = high-intensity functional training; WL = weightlifting; PL/Plyo = plyometric training; VJ = vertical jump training; CG/C = control group; KB = kettlebell training; WT/TW = weight/traditional weight training; OL = Olympic weightlifting; WP = combined weightlifting and plyometric training; CATCH = catching-derivative training; PULL = pulling-derivative training; PC = power clean; CP = clean pull; RM = repetition maximum; 1RM = one-repetition maximum

**Appendix S3. Node-Splitting Analysis Results**

The node-splitting analysis results for CMJ height, CMJ power, SJ height, and SJ power are shown in Tables S2–S5. No statistically significant inconsistency was observed between direct and indirect evidence for any comparison (all *P* > 0.05), indicating that the consistency assumption was satisfied and supporting the use of the consistency model in the network meta-analysis.

**CMJ-Height**

The results of the node-splitting analysis assessing consistency between direct and indirect evidence for CMJ height are presented in Table S2.

**Table S2.** Node-splitting results for CMJ-Height

| **Comparison** | **Direct_MD** | **Direct_SE** | **Indirect_MD** | **Indirect_SE** | **Z** | **P_value** | **Direct_studies** |
| --- | --- | --- | --- | --- | --- | --- | --- |
| Control vs COMB | -4.400 | 3.406 |  |  |  |  | Fotini 2010 |
| OW-related vs COMB | 0.200 | 3.540 |  |  |  |  | Fotini 2010 |
| PLYO vs COMB | -3.500 | 3.456 |  |  |  |  | Fotini 2010 |
| OW-related vs Control | 2.426 | 1.206 |  |  |  |  | Fotini 2010; Fotini2012; Ricardo 2022; Valmon 2005 |
| PLYO vs Control | 0.706 | 1.294 | -4.527 | 3.901 | 1.273 | 0.203 | Fotini 2010; Ricardo 2022; Valmon 2005 |
| RT vs Control | -1.820 | 2.283 | 0.902 | 1.682 | -0.960 | 0.337 | Fotini2012 |
| PLYO vs OW-related | -2.175 | 1.029 |  |  |  |  | Fotini 2010; Ricardo 2022; Sheldon 2009; Valmon 2005 |
| RT vs OW-related | -2.441 | 1.018 |  |  |  |  | Fotini2012; Sheldon 2009; William 2012 |
| RT vs PLYO | 2.000 | 4.494 | -0.828 | 1.410 | 0.600 | 0.548 | Sheldon 2009 |

OW-related = Olympic weightlifting and weightlifting-derivative training; PLYO = plyometric or jump training; RT = non-Olympic-weightlifting resistance training; COMB = combined or complex training.

**CMJ-Power**

The results of the node-splitting analysis assessing consistency between direct and indirect evidence for CMJ power are presented in Table S3.

**Table S3.** Node-splitting results for CMJ-Power

| **Comparison** | **Direct_MD** | **Direct_SE** | **Indirect_MD** | **Indirect_SE** | **Z** | **P_value** | **Direct_studies** |
| --- | --- | --- | --- | --- | --- | --- | --- |
| Control vs COMB | 5.191 | 5.141 |  |  |  |  | Fotini 2010 |
| OW-related vs COMB | 2.255 | 3.660 |  |  |  |  | Fotini 2010 |
| PLYO vs COMB | 1.071 | 3.374 |  |  |  |  | Fotini 2010 |
| OW-related vs Control | 0.418 | 1.168 |  |  |  |  | Fotini 2010; Fotini2012; Ricardo 2022 |
| PLYO vs Control | 1.025 | 1.861 |  |  |  |  | Fotini 2010; Ricardo 2022 |
| RT vs Control | -3.000 | 1.141 |  |  |  |  | Fotini2012 |
| PLYO vs OW-related | -0.173 | 1.707 |  |  |  |  | Fotini 2010; Ricardo 2022 |
| RT vs OW-related | -2.800 | 1.357 |  |  |  |  | Fotini2012 |

OW-related = Olympic weightlifting and weightlifting-derivative training; PLYO = plyometric or jump training; RT = non-Olympic-weightlifting resistance training; COMB = combined or complex training.

**SJ-Height**

The results of the node-splitting analysis assessing consistency between direct and indirect evidence for SJ height are presented in Table S4.

**Table S4.** Node-splitting results for SJ-Height

| **Comparison** | **Direct_MD** | **Direct_SE** | **Indirect_MD** | **Indirect_SE** | **Z** | **P_value** | **Direct_studies** |
| --- | --- | --- | --- | --- | --- | --- | --- |
| Control vs COMB | -2.600 | 2.824 |  |  |  |  | Fotini 2010 |
| OW-related vs COMB | 0.100 | 2.948 |  |  |  |  | Fotini 2010 |
| PLYO vs COMB | 0.200 | 3.502 |  |  |  |  | Fotini 2010 |
| OW-related vs Control | 2.643 | 1.096 |  |  |  |  | Fotini 2010; Fotini2012; Ricardo 2022; Valmon 2005 |
| PLYO vs Control | 0.880 | 1.219 | -6.212 | 3.744 | 1.801 | 0.072 | Fotini 2010; Ricardo 2022; Valmon 2005 |
| RT vs Control | -1.300 | 1.892 | -2.718 | 3.294 | 0.373 | 0.709 | Fotini2012 |
| PLYO vs OW-related | -2.787 | 1.025 |  |  |  |  | Fotini 2010; Ricardo 2022; Sheldon 2009; Valmon 2005 |
| RT vs OW-related | -4.909 | 1.730 |  |  |  |  | Fotini2012; Sheldon 2009 |
| RT vs PLYO | 3.000 | 4.025 | -1.904 | 1.824 | 1.110 | 0.267 | Sheldon 2009 |

OW-related = Olympic weightlifting and weightlifting-derivative training; PLYO = plyometric or jump training; RT = non-Olympic-weightlifting resistance training; COMB = combined or complex training.

**SJ-Power**

The results of the node-splitting analysis assessing consistency between direct and indirect evidence for SJ power are presented in Table S5.

**Table S5.** Node-splitting results for SJ-Power.

| **Comparison** | **Direct_MD** | **Direct_SE** | **Indirect_MD** | **Indirect_SE** | **Z** | **P_value** | **Direct_studies** |
| --- | --- | --- | --- | --- | --- | --- | --- |
| Control vs COMB | -8.018 | 3.073 |  |  |  |  | Fotini 2010 |
| OW-related vs COMB | -4.013 | 2.992 |  |  |  |  | Fotini 2010 |
| PLYO vs COMB | -5.677 | 2.818 |  |  |  |  | Fotini 2010 |
| OW-related vs Control | 2.437 | 1.341 |  |  |  |  | Fotini 2010; Fotini2012; Ricardo 2022 |
| PLYO vs Control | 2.377 | 1.740 |  |  |  |  | Fotini 2010; Ricardo 2022 |
| RT vs Control | 4.052 | 1.503 |  |  |  |  | Fotini2012 |
| PLYO vs OW-related | -1.131 | 1.493 |  |  |  |  | Fotini 2010; Ricardo 2022 |
| RT vs OW-related | 3.568 | 2.369 |  |  |  |  | Fotini2012 |

OW-related = Olympic weightlifting and weightlifting-derivative training; PLYO = plyometric or jump training; RT = non-Olympic-weightlifting resistance training; COMB = combined or complex training.

**Appendix S4. SUCRA Analysis Results**

To further evaluate the relative ranking of the interventions across outcomes, SUCRA values for CMJ height, CMJ power, SJ height, and SJ power are presented in Table S6.

**Table S6.** SUCRA values for CMJ height, CMJ power, SJ height and SJ power.

| **Outcome** | **OW-related** | **PLYO** | **RT** | **COMB** | **Control** |
| --- | --- | --- | --- | --- | --- |
| CMJ-Height | 82.4 | 33.7 | 22.3 | 81.6 | 30.0 |
| CMJ-Power | 72.0 | 71.5 | 10.0 | 37.1 | 59.4 |
| SJ-Height | 90.2 | 40.8 | 10.5 | 71.9 | 36.6 |
| SJ-Power | 46.0 | 31.6 | 73.0 | 94.7 | 4.7 |

SUCRA = surface under the cumulative ranking curve; CMJ = countermovement jump; SJ = squat jump; OW-related = Olympic weightlifting and weightlifting-derivative training; PLYO = plyometric or jump training; RT = non-OW resistance training; COMB = combined or complex training.

SUCRA values were calculated from the rank probability distributions to summarize the relative ranking of each intervention node. For CMJ height, OW-related training had the highest SUCRA value (82.4%), followed closely by COMB (81.6%). For CMJ power, OW-related training (72.0%) and PLYO (71.5%) showed the highest SUCRA values. For SJ height, OW-related training ranked highest (90.2%), followed by COMB (71.9%). For SJ power, COMB had the highest SUCRA value (94.7%), followed by RT (73.0%). Given the small and sparse evidence networks, these SUCRA values were interpreted as exploratory ranking metrics rather than definitive evidence of intervention superiority.

**Reference**

1. Berton R, Silva D, Lopes dos Santos M, Silva C, Tricoli V. Weightlifting derivatives vs. plyometric exercises: Effects on unloaded and loaded vertical jumps and sprint performance. PLOS ONE. 2022;17:e0274962. https://doi.org/10.1371/journal.pone.0274962

2. Otto W, Coburn J, Brown L, Spiering B. Effects of Weightlifting vs. Kettlebell Training on Vertical Jump, Strength, and Body Composition. J Strength Cond Res Natl Strength Cond Assoc. 2012;26:1199–202. https://doi.org/10.1519/JSC.0b013e31824f233e

3. Posnakidis G, Aphamis G, Giannaki CD, Mougios V, Bogdanis GC. The Addition of High-Load Resistance Exercises to a High-Intensity Functional Training Program Elicits Further Improvements in Body Composition and Strength: A Randomized Trial. Sports Basel Switz. 2022;10:207. https://doi.org/10.3390/sports10120207

4. Suchomel T, McKeever S, McMahon J, Comfort P. The Effect of Training with Weightlifting Catching or Pulling Derivatives on Squat Jump and Countermovement Jump Force–Time Adaptations. J Funct Morphol Kinesiol. 2020;5. https://doi.org/10.3390/jfmk5020028

5. Arabatzi F, Kellis E, Sáez de Villarreal E. Vertical Jump Biomechanics after Plyometric, Weight Lifting, and Combined (Weight Lifting + Plyometric) Training. J Strength Cond Res Natl Strength Cond Assoc. 2010;24:2440–8. https://doi.org/10.1519/JSC.0b013e3181e274ab

6. Arabatzi F, Kellis E. Olympic Weightlifting Training Causes Different Knee Muscle–Coactivation Adaptations Compared with Traditional Weight Training. J Strength Cond Res Natl Strength Cond Assoc. 2011;26:2192–201. https://doi.org/10.1519/JSC.0b013e31823b087a

7. Hawkins S, Doyle T, McGuigan M. The Effect of Different Training Programs on Eccentric Energy Utilization in College-Aged Males. J Strength Cond Res Natl Strength Cond Assoc. 2009;23:1996–2002. https://doi.org/10.1519/JSC.0b013e3181b3dd57

8. Tricoli V, Lamas L, Carnevale R, Ugrinowitsch C. SHORT-TERM EFFECTS ON LOWER-BODY FUNCTIONAL POWER DEVELOPMENT: WEIGHTLIFTING VS.VERTICAL JUMP TRAINING PROGRAMS. J Strength Cond Res. 2005;19:433.
